# Emerging Molecular Pathways Governing Dietary Regulation of Neural Stem Cells during Aging

**DOI:** 10.3389/fphys.2017.00017

**Published:** 2017-01-30

**Authors:** Chiara de Lucia, Tytus Murphy, Sandrine Thuret

**Affiliations:** Neurogenesis and Mental Health Laboratory, Department of Basic and Clinical Neuroscience, Institute of Psychiatry, Psychology and Neuroscience, King's College LondonLondon, UK

**Keywords:** nutrients, diet, aging, mTOR, sirtuins, insulin-like growth factor signaling, IIS, epigenetics

## Abstract

Aging alters cellular and molecular processes, including those of stem cells biology. In particular, changes in neural stem cells (NSCs) are linked to cognitive decline associated with aging. Recently, the systemic environment has been shown to alter both NSCs regulation and age-related cognitive decline. Interestingly, a well-documented and naturally occurring way of altering the composition of the systemic environment is through diet and nutrition. Furthermore, it is well established that the presence of specific nutrients as well as the overall increase or reduction of calorie intake can modulate conserved molecular pathways and respectively reduce or increase lifespan. In this review, we examine these pathways in relation to their function on NSCs and cognitive aging. We highlight the importance of the Sirtuin, mTOR and Insulin/Insulin like growth factor-1 pathways as well as the significant role played by epigenetics in the dietary regulation of NSCs and the need for further research to exploit nutrition as a mode of intervention to regulate NSCs aging.

## Aging and neural stem cell function

Aging is the number one risk factor for the majority of diseases currently affecting the developed and developing world (Niccoli and Partridge, 2012). The area of study addressing this issue, known as biogerontology, is committed to investigating the underlying mechanisms of aging, to explore whether they can be intervened upon to delay or perhaps even halt the progression of age-related conditions such as cardiovascular disease, cancer and neurodegeneration (Verburgh, 2015). An improved understanding of aging mechanisms could lead to the development of strategies to increase “healthspan”—the period of time free from debilitating disease (Franklin and Tate, 2008; Brandhorst et al., 2015). Aging is linked to a number of cellular and molecular processes including nutrient-sensing pathway, epigenetic and stem cells biology deregulation (López-Otín et al., 2013). Stem cells in general have been closely linked to aging owing to their reduced regenerative ability linked to the decline of tissues that accompanies age (Signer and Morrison, 2013; Behrens et al., 2014). Impaired function of satellite stem cells in muscle and epidermal stem cells of the skin, for example, is a key process underlying reduced regeneration during aging in these tissues (Castilho et al., 2009; Day et al., 2010).

The adult NSC population is also negatively affected by age. Post-natal NSCs, able to differentiate into neurons, have been identified in several areas of the mammalian central nervous system (CNS), including the rodent and human dentate gyrus (DG) of the hippocampus and the mouse subventricular zone (SVZ). Postnatal-born neurons—a phenomenon known as adult neurogenesis—in these areas have been implicated in learning and memory and olfaction respectively (Altman and Das, 1965; Pencea et al., 2001; Spalding et al., 2013). More recently post-natal NSC-derived neurons have also been identified in the mouse hypothalamus and the human striatum (Kokoeva et al., 2005; Ernst et al., 2014). Though mouse models have been extensively used in adult neurogenesis research, it is becoming clear that there are considerable differences between rodent and human neurogenesis: firstly, there seems to be no neuron turnover in the human olfactory bulb, whilst this is an important neurogenic zone in the mouse brain. Secondly, whilst hippocampal neurogenesis occurs in both species, retrospective birth dating of human postmortem tissue has revealed that nearly all of the dentate granule “turnover” during adult life, whereas only 10% of the mouse granule neurons are exchanged in adulthood (Spalding et al., 2013). Furthermore, the rate of decline in hippocampal neurogenesis in response to age appears far greater in mice than in humans (Bergmann et al., 2015).

**Figure 1:**
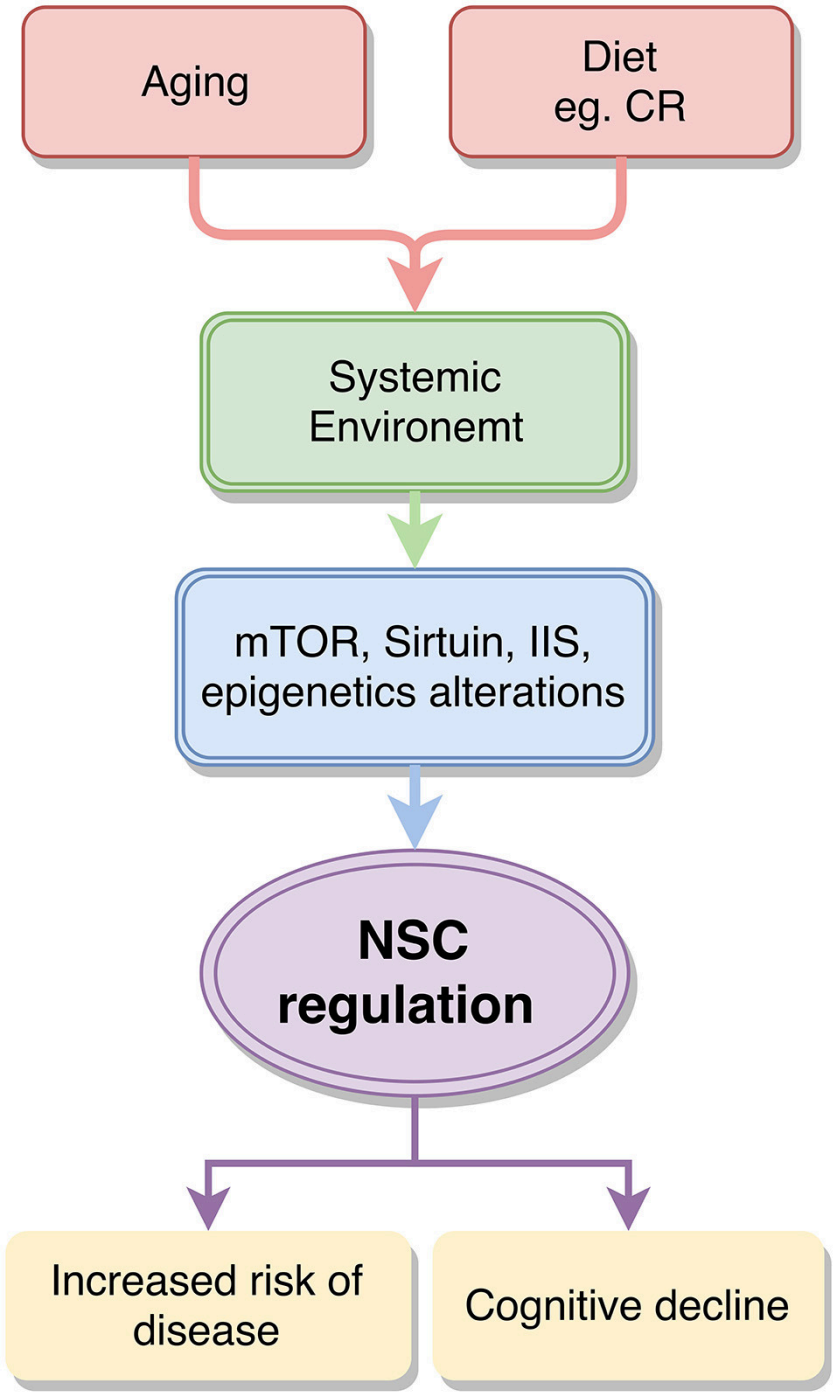
**Diagram summarizing the proposed relationship between aging, nutrition and neural stem cells**. Aging and dietary modulation such as calorie restriction (CR) can alter the composition of the systemic environment, which affects epigenetic regulation and several nutrient-sensing pathways, including the mTOR, Sirtuin and Insulin and Insulin like signaling (IIS) pathways. These in turn alter neural stem cell (NSC) regulation and can predispose to disease and cognitive decline.

The marked decline in NSC activity in the aging rodent DG manifests in reduced proliferation that eventually leads to the depletion of the progenitor pool (Romine et al., 2015; Yang et al., 2015). This decline partly contributes to the age-linked decline in cognitive abilities, particularly those dependent on the hippocampus (Park et al., 2013; Romine et al., 2015; Yang et al., 2015). In the SVZ, there is a similar marked decline in NSC function and this contributes to impoverished olfaction during rodent aging (Enwere et al., 2004). Studies in model species have shown that deregulated hippocampal neurogenesis, is an important component of neurodegenerative conditions such as Alzheimer's (López-Toledano and Shelanski, 2004, 2007; Winner et al., 2011), further linking declines in NSC function to the deterioration of the aging brain. Aging therefore appears to exert some of its detrimental effects in the CNS by directly interfering with multiple cellular and molecular processes that govern the regulation of the NSC population. This notion is summarized in Figure 1. As aging has such severe effects on the decline of NSC and the development of neurodegenerative conditions, this prompts the field to consider whether we can we target the maintenance of the NSC population to slow cognitive aging and neurodegenerative disease progression? If so, what are the key mechanisms to target?

Interestingly, studies investigating the role of aging on NSCs have also highlighted the importance of the systemic milieu in regulating the neurogenic niches of the CNS; through the use of heterochronic parabiosis, Villeda and colleagues showed that the systemic environment of a young mouse was able to rescue the cognitive deficits of an aged mouse following the fusion of young-old circulatory systems (Villeda et al., 2011, 2014). This rejuvenating effect is also observed following intravenous infusion of young plasma into old mice, and was underpinned by a marked reversal of age-related decline in hippocampal neurogenesis (Villeda et al., 2011) and upregulation of genes linked to synaptic plasticity (Villeda et al., 2014). Similarly, enhanced neurogenesis in the SVZ was observed by Katsimpardi and colleagues following heterochronic parabiosis, resulting in improved odor discrimination (Katsimpardi et al., 2014). Notably, these studies have demonstrated the role for candidate chemokines (e.g., CCL11, Villeda et al., 2011) and growth factors (e.g., GDF11 e.g., Loffredo et al., 2013) whose circulating levels fluctuate in aging mice and appear to exert their effects, at least in part, by altering NSC function. Complementary to this, studies looking at other populations of stem cells have observed a similarly rejuvenating effect of the youthful milieu on the typical age-related declines in stem cell function throughout the body as well as vice-versa, whereby the old milieu inhibited stem cell function (Conboy and Rando, 2012). These data suggest that by altering the composition of the systemic environment, one could affect the regulation of the NSCs; a well-documented, naturally occurring way of achieving this is through diet and nutrition (Stangl and Thuret, 2009). We define diet as encompassing dietary paradigms such as calorie restriction (CR), intermitted fasting (IF), dietary restriction (e.g., of specific components such as protein) (DR) and time restricted feeding (TRF) whereas nutrition involves the intake and/or supplementation of foods containing pro-neurogenic agents such as polyphenols, polyunsaturated fatty acids (PUFAs) and vitamins/minerals.

As stem cells in general are designed to act in response to their environment and, and as reported above, are responsive to differing compositions of the systemic environment, it follows that diet can greatly influence their function (Murphy and Thuret, 2015). Several authors have reviewed the effect of diet on stem cells in general (Rafalski et al., 2012; Ochocki and Simon, 2013; Mihaylova et al., 2014), for the purpose of this review we will focus on the relatively unexplored field of the effects of diet and aging on NSCs. Briefly, nutritionists have found that an overabundance of nutrients is detrimental to several aspects of human and animal health (Keenan et al., 1994; Nagai et al., 2012). This results from an overstimulation of nutrient sensing molecular pathways, which eventually become insensitive to the stimuli (Blagosklonny, 2008; Gems and de la Guardia, 2013). CR, IF and DR have been reported to have opposite effects on these pathways and present a means to improved health and life span (Solon-Biet et al., 2014, 2015; Brandhorst et al., 2015; Fontana and Partridge, 2015). Figure 1 is a schematic of how aging, diet and the systemic environment interact to act upon these pathways. Notably, these effects are conserved across species as they have been observed in simple organisms such as yeast, *C. elegans*, and drosophila through to rodents and humans (Fontana et al., 2010). We now review the impacts of dietary and nutritional regulation of NSC activity and function, with focus on the aforementioned nutrient sensing pathways and their effects on longevity.

## Impact of diet on neural stem cells

Dietary paradigms such as CR and IF are the most widely employed means of assessing the impacts of diet upon stem cell function and longevity. A 30–40% reduction in calorie intake, the typical regimen for CR, has often been brought about by alternate-day feeding—a form of IF. As such, the field must pay great attention as to whether they are assessing the impacts of DR or IF, unpublished work from our lab shows that the positive impacts of IF on hippocampal neurogenesis and cognitive performance can be derived independent of calorie intake, suggesting that it is the period of fasting that that is acting on the NSC pool. Nutritional content, such as the amount of polyphenols and PUFAs, among other specific nutrients, has also been reported to impact on NSC function during aging (see Maruszak et al., 2014; Murphy and Thuret, 2015 for review).

Reducing calorie intake in rodents was shown to counteract age-related cognitive decline as well as increase the number of newly generated neurons in the hippocampus (Ingram et al., 1987; Lee et al., 2000, 2002). Furthermore, Kumar and colleagues suggest DR may aid in fighting excitotoxic injury as an increase in progenitors is seen in the SVZ, SGL, hypothalamus and cortex of adult rats (Kumar et al., 2009). In contrast, Bondolfi and colleagues found that CR did not affect the rate of neurogenesis but affected the survival of new–born glia in the mouse hippocampus (Bondolfi et al., 2004). Given that CR and IF (typically involving some degree of CR) have been shown to positively impact on NSC function, it follows that excessive calorie intake, as in the case of obesity and other models of metabolic disorders, will negatively affect NSC activity and may decrease adult hippocampal neurogenesis (Stangl and Thuret, 2009). Furthermore, obesity was a detrimental factor in studies investigating post-stroke recovery in humans, suggesting that a history of increased calorie intake impairs brain repair (Kalichman et al., 2007). Interestingly, CR was also proven beneficial in elderly humans as shown by improved verbal memory scores (Witte et al., 2009). It must be considered however, that in elderly, sometimes frail individuals, restricting of calories may be too dangerous, older populations may therefore benefit from more targeted pharmacological interventions to modulate NSCs activity based on CR/IF and DR mimetics.

Though it may seem counter intuitive that reducing calories may be beneficial to stem cells in particular, this can be explained by our historical food supply not being readily available and abundant at all times. Humans have evolved to cope with periods of reduced calorie intake, resembling the effect achieved by CR and IF experiments. A possible biological explanation for this relies on the benefits of refeeding after a fasting period, suggesting that when an organism is “fasting” it can focus on preparing resources to act quickly and effectively when nutrients do become available (Reed et al., 1996). With respects to the NSCs of the DG in particular, their activity is possibly enhanced in the absence of nutrients owing to the necessity of “hunting behavior” and the requirement for cognitive flexibility that must accompany it: improved cognition may be a means to ensure food is found (As discussed by Mattson, 2012).

## Supporting evidence for the role of nutrient-sensing pathways in neural stem cell regulation and longevity

**Figure 2:**
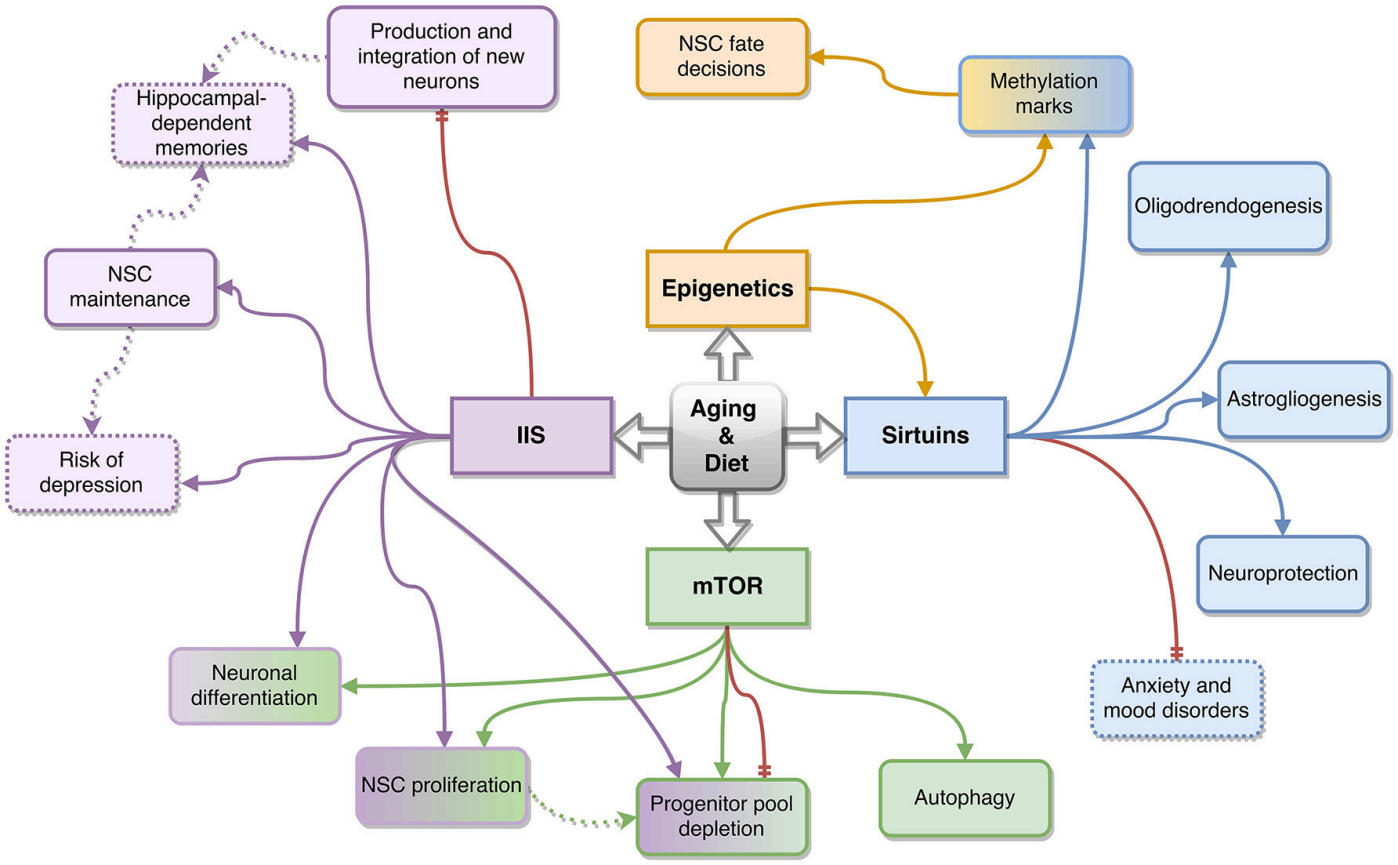
**Schematic summarizing the key effects of the Insulin/Insulin-like Growth factor (IIS) (in purple), mTOR (in green), sirtuin (in blue) and epigenetic (in orange) pathways on neural stem cell (NSC) function as a result of diet and aging**. Solid arrows represent relationships between the molecular pathways (represented by the four rectangles in the center) and NSC function (represented by the outer, rounded rectangles) or related phenotypes (dotted rounded rectangles) discussed in this review. The dotted arrows connect possible molecular mechanisms behind the observed phenotypes reported in this review. Red arrows represent an effect caused by the inhibition of the pathway they stem from. Boxes with gradient coloring represent functions affected by multiple pathways.

Owing to the compelling research relating diet to longevity and to NSCs, the field is now trying to delineate the molecular pathways behind this relationship. Though many pathways are involved in NSC regulation and an equally vast amount is involved in nutrient-sensing and aging, there is a relatively small proportion identified as relating the three. Thus far, the best characterized, and therefore most promising starting point for imminent studies, are the mTOR, Insulin and Insulin-like growth factor signaling and Sirtuin pathways. In this section, we will focus on the available data supporting a role for these pathways in aging, nutrient sensing and NSC regulation. See Figure 2 for a summary of key NSC related functions affected by these pathways.

### mTOR

The mammalian target of rapamycin (mTOR) is a classical dietary and nutrient sensing pathway. mTOR is a serine/threonine kinase. It combines with several accessory proteins including RAPTOR or RICTOR to form mTOR complex 1 (mTORC1) and mTOR complex 2 (mTORC2), respectively. The two complexes carry out different functions with mTORC1 being responsible for cell growth and metabolism while mTORC2 regulates cytoskeleton organization. Importantly, mTOR is the catalytic subunit of the complex and is inhibited by the presence of rapamycin, this inhibition however only occurs when mTOR is bound to the RAPTOR protein (Magri and Galli, 2013). mTOR activity is altered by energy and amino acid availability as well as growth factors, making it a key molecule within nutrition studies (Magri and Galli, 2013). mTOR is also involved in a feedback loop with insulin where insulin (discussed below) activates mTOR and mTOR phosphorylates the S6 kinase, which in turn inhibits insulin signaling (Blagosklonny, 2008). In the liver, branched chain amino acids (BCAA) in particular signal for mTOR activation, showing that protein intake and specific amino acids alter the total activation of mTOR (measured as phosphorylated mTOR / total mTOR) and ultimately longevity (Solon-Biet et al., 2014).

As well as functions in nutrient sensing, the mTOR pathway has also been linked to longevity (Blagosklonny, 2010). Its inhibition via ethylaxanthine and rapamycin was shown to improve longevity in yeast (Wanke et al., 2008) and mice (Harrison et al., 2009; Fok et al., 2014) respectively, leading to plans to test the compound in larger and longer-lived mammals such as companion dogs (Check Hayden, 2014). Recently, the mechanism of action behind rapamycin's role in longevity was suggested to involve a decrease in reactive oxygen species, as shown by experiments in the rodent liver (Martínez-Cisuelo et al., 2016). Notably, rapamycin treatment was shown to exert its beneficial effects even if started at advanced age, a factor, which usually greatly impairs the efficacy of life-extending interventions (Harrison et al., 2009). In addition, Tan and colleagues highlighted increased PI3K/Akt/mTOR pathway activation during aging as the molecular mechanism responsible for replicative senescence in vascular smooth muscle cells (Tan et al., 2016). This pathway has been the focus of aging research and is shared by both the mTOR and the insulin-like growth factor signaling (IIS) pathways as explained in Section Insulin. Several consequences of mTOR activation increase the risk of premature aging and disease, these include decreased autophagy accompanied by an increase in protein production eventually causing an increase in protein agglomeration as well as an increase in inflammation (Verburgh, 2015). Furthermore, chronic mTOR activation causes increased proliferation of several types of stem cells eventually leading to progenitor pool depletion. Adequate mTOR regulation could thus be key in maintaining these populations throughout aging (Sato et al., 2010; Paliouras et al., 2012).

Though there is a limited number of studies investigating the role of mTOR within the NSC population, there have been several encouraging findings supporting key functions for this nutrient sensing pathway in NSC regulation and aging.

Firstly, both protein deposition and inflammation are key hallmarks of neurodegenerative conditions suggesting that mTOR hyperactivity may contribute to disease progression within the CNS (O' Neill, 2013). These observations are consistent with the evidence supporting aging as a consequence of overstimulation of signaling pathways, driven by the overabundance of nutrients. Furthermore, mTOR has also been shown to be key in dictating the proliferation rate of the adult SVZ NSC population; its inhibition in fact, caused progenitor pool depletion (Paliouras et al., 2012). In addition, Han and colleagues have also reported that, during embryonic development, mTOR carries an important role for neuronal differentiation: enhancing mTOR activity via insulin caused an increase in the number of neurons, which was counteracted by rapamycin. Intriguingly, in the presence of rapamycin the decrease in neuronal numbers was attributed to increased autophagy rather than apoptosis (Han et al., 2008). These findings also suggested the possibility of similar mechanisms taking place in adult NSCs. Indeed, Yu and colleagues showed that increased autophagic death also occurred in adult hippocampal NSC following insulin withdrawal and was accompanied by a decrease in cell density, these effects were also exacerbated by the presence of rapamycin, implicating mTOR activity (Yu et al., 2008). The authors state that in an aged environment there is decreased insulin signaling and thus increased autophagy, which may reduce the survival of stem cells. Contrary to this, the authors remark that autophagy can enhance cell survival, given its beneficial effect in clearing unwanted and malfunctioning cells, an essential mechanism in the context of aging (Cuervo, 2008). Elevated autophagy in response to reduced insulin signaling may thus not be a solely negative effect but further research is required to better delineate the modulatory role of mTOR activity in NSC during aging (Gems and de la Guardia, 2013).

The limited number of studies (See Table 1) however, highlights the need for further investigations into the exact mechanisms of mTOR regulation in adult NSCs, with the ultimate aim of determining whether modulation of this pathway can bring preserve the NSC pool during aging. The key effects of the mTOR pathway on NSCs elucidated this far are depicted in Figure 2.

**Table 1:** **Table summarizing studies showing supporting evidence for the role of mTOR, IIS, and Sirtuin pathways in NSCs function**.

**Pathway**	**Model**	**NSC**	**Intervention**	**Supporting evidence**	**Study**
mTOR/IIS	Rat *In vitro*	Embryonic	Increased insulin levels	Increase in differentiated neurons, which was counteracted by rapamycin	Han et al., 2008
mTOR/IIS	Rat *In vitro*	DG	Insulin withdrawal	Increased neuronal death, exacerbated by rapamycin	Yu et al., 2008
IIS	Mouse *In vitro*	SVZ and DG	*FoxO3* knockout	Decreased number of NSC and self-renewal ability	Renault et al., 2009
mTOR/IIS	Rat *In vivo*	Cortex	Treated with EGCG + TBI	Reduced NSC cell death around damaged area	Itoh et al., 2012
IIS	Rat *In vitro*	DG	Increased IGF-1	Decreased differentiation and increased proliferation of NSCs	Åberg et al., 2003
IIS	Mouse *In vitro*	Striatal	Increased insulin	Increases NSCs differentiation	Arsenijevic et al., 2001
IIS	Mouse *In vivo*	Perinatal	IGF-1 overexpression	Increase in number of neurons and of oligodendrocytes.	Carson et al., 1993
IIS	Mouse *In vivo*	Perinatal	*IGF-1* KO	Decreased proliferation and differentiation of oligodendrocytes	Ye et al., 2002
IGF-1	Mouse *In vivo*	SVZ	IGF-1R KO	Reduced age related depletion of NSC	Chaker et al., 2015
IGF-II	Mouse *In vitro*	Perinatal	IGF-II treatment	Increased NSC expansion and promoted self-renewal	Ziegler et al., 2012
IGF-II	Mouse *In vitro*	DG	Sh-RNA knockdown of *IGF-II*	Impaired proliferation	Bracko et al., 2012
Sirtuins	Mouse *In vitro*	Perinatal SVZ	Oxidation or Sirt1 activation	Enhanced astrocytic lineage	Prozorovski et al., 2008
Sirtuins	Mouse *In vitro*	Perinatal SVZ	Reducing environment	Enhanced neuronal lineage	Prozorovski et al., 2008
Sirtuins	Mouse *In vitro*	Perinatal SVZ	Sh-RNA knockdown of *Sirt1*	Disengaged neural fate from redox conditions	Prozorovski et al., 2008
Sirtuins	Mouse *In vivo*	SVZ and DG	Inactivation of *Sirt1*	Increased oligodendrocyte differentiation and myelination	Rafalski et al., 2013
Sirtuins/NAMPT	Mouse *In vivo*	DG	Measuring /ablating NAMPT	NAMPT levels decrease with age, its ablation reduces NSC proliferation and oligodendrogenesis	Stein and Imai, 2014
Epigenetics	Mouse *In vitro*	Embryonic	*Dnmt1* knockout	Increased astrocytic differentiation	Fan et al., 2005
Epigenetics	Mouse *In vitro*	Perinatal SVZ	*Dnmt3* knockout	Impaired neuronal differentiation	Wu et al., 2010

*NSC, neural stem cells, IIS, insulin and insulin-like signaling, DG, dentate gyrus, SVZ, sub ventricular zone, KO, knockout*.

### Insulin

Insulin and Insulin-like growth factor (IGF) are two hormones closely linked to nutrition, as they respond to increased glucose. Insulin is released by the β cells of the pancreas and acts upon transmembrane tyrosine kinase receptor to activate downstream signaling pathways and cause glucose uptake by liver and muscle cells (van Heemst, 2010). Other members of the IGF family are under the control of growth hormone (GH) release by the pituitary gland. They also act upon tyrosine kinase receptors and activate downstream pathways that are often shared with insulin, coining the term insulin/insulin-like growth factor signaling (IIS) pathway. Stimulation of the IIS pathway results in the activation of the PI3K/Akt pathway, a pathway shared with mTOR signaling, which ultimately leads to the inactivation of the FoxO transcription factors (van Heemst, 2010). While both insulin and IGF respond to carbohydrate presence, they usually carry out slightly different roles with insulin being primarily occupied with glucose metabolism and IGF with growth and survival (Rafalski and Brunet, 2011). Besides being activated by carbohydrates such as glucose, the IIS pathway is also stimulated by proteins and is involved in a feedback loop with mTOR as described in Section mTOR (Blagosklonny, 2008).

IIS is also extensively implicated in longevity (Kimura et al., 1997; Bartke et al., 2013); overactivation, like for mTOR, leads to decreases autophagy and ultimately to the shortening of lifespan (Verburgh, 2015). The decrease of the IIS pathway in C. elegans by Kimura and colleagues was one of the first experiments to show an increase in lifespan relating to diminished insulin signaling (Kimura et al., 1997). This was confirmed by several other studies, with Blüher and colleagues showing that the knock-out of a fat specific insulin receptor resulted in an increase in lifespan of 18% (Blüher et al., 2003) and Taguchi et al finding that the knock-out of downstream substrates of the IIS pathway caused a 32% increase in female mice lifespan (Taguchi et al., 2007). Furthermore, serum IGF-1 levels in 31 different mouse strains negatively correlated to average lifespan (Yuan et al., 2009). Some encouraging evidence has also been found relating IIS to human longevity; IIS related polymorphisms correlate to lifespan (Bonafè et al., 2003; Kojima et al., 2004) and several centenarians were found to have loss of function mutations in the IGF-1 receptors (Suh et al., 2008). Conversely, people affected by acromegaly, characterized by increased GH release, experienced a 2–3 fold increase in death rate (Clayton, 2003; Suh et al., 2008; Verburgh, 2015). More recently, reduced growth hormone secretion, and thus indirectly IIS activity, was shown to correlate with human familial longevity (van der Spoel et al., 2016) and genomewide meta-analysis studies linked several gene loci to longevity and to levels of circulating IGF-related proteins (Teumer et al., 2016). These studies show that this pathway and its role in lifespan is conserved in more complex organisms.

Several rodent studies have suggested insulin and IGF may link nutrition to organism longevity through key functions in tissue-specific stem cell maintenance. Mechanisms such as autophagy, compromised by IIS overstimulation, carry out key functions within the stem cell population. FoxO3A was proven essential for efficient clearing of age-related cellular debris which is known to prevent malfunctioning of stem cells and to lead to improved longevity (Cuervo, 2008; Warr et al., 2013). The IIS pathway also appears to have important functions on the regulation of neural stem cells specifically, with studies implicating it in both development and adulthood.

IGF-1 overexpression alone and IGF-1 and insulin overexpression on embryonic NSC for example, highlighted that insulin pushes toward increased differentiation while IGF-1 pushes toward a proliferative phenotype (Arsenijevic et al., 2001). Furthermore, studies have shown that overexpression of IGF-1 leads to increased brain size due to increased myelination (Carson et al., 1993). Finally, *Igf-1* null mice presented with decreased proliferation and differentiation of oligodendrocyte lineage (Ye et al., 2002).

Whether these effects persist into adulthood requires further investigation. Recently however, Chaker and colleagues showed that the inhibition of IGF-1 signaling in rodent adult olfactory bulb NSCs was able to hinder age-related stem cell decline and preserve the production and integration of newborn neurons (Chaker et al., 2015). IGF-1 was also shown to stimulate proliferation of adult hippocampal NSC both *in vivo* and *in vitro* while blocking the PI3K/Akt pathway stopped the proliferative effects of IGF-1 on NSCs (Åberg et al., 2000, 2003). This was later supported by Chigogora and colleagues finding a correlation between IGF-1 levels and an elevated risk of human depression (Chigogora et al., 2016), a disorder known to involve neurogenic and possible NSC deregulation (Hill et al., 2015). Furthermore, the deletion of the FoxO family members results in increased brain size and proliferation during development but also in a depletion of the progenitor pool and ultimately a decrease of SVZ adult neurogenesis (Paik et al., 2009; Renault et al., 2009). FoxO3 in particular seems to regulate quiescence of the adult SGL and SVZ NSC population and to have a role in oligodendrocyte regulation. FoxO transcription factors are also sensitive to oxygen changes making them ideal effectors between oxidative stress, a known aspect of aging, and stem cell maintenance (Renault et al., 2009). Besides these studies, several others have reported pro-neurogenic effects of insulin when investigating the mTOR pathway as discussed in Section mTOR.

Interestingly, IGF-II is produced by choroid plexus and released in the cerebrospinal fluid (CSF), allowing it to come in contact with the neurogenic niches. NSCs in the SVZ extend a process through the ventricular wall and come in contact with the CSF directly, thereby allowing its composition to directly alter their regulation. Increased presence of CSF IGF-II during development for example, promotes neurogenesis (Lehtinen et al., 2011; Ziegler et al., 2015). IGF-II is also involved in hippocampal neurogenesis in adulthood (Bracko et al., 2012). *In vitro* and *in vivo* studies showed IGF-II involvement in promoting NSC maintenance (Ziegler et al., 2012). Studies have also linked IGF-II dependent mechanisms to hippocampal-dependent memory retention (Chen et al., 2011) and more specifically to age-related cognitive decline (Steinmetz et al., 2016) in rodents, further supporting a link between IGF-II and NSCs function and presenting a key target for further researching seeking to preserve AHN during aging (See Table 1).

The overall effects of this pathway on NSC regulation however, remain inconclusive due to a limited number of concordant studies (Åberg et al., 2003; Itoh et al., 2012); some studies have reported increased IIS resulting in a beneficial increase in adult neurogenesis, for example during GH and IGF-1 mediated increases in neurogenesis as a result of exercise (Berg and Bang, 2004) or following a blueberry supplemented diet in rodents (Shukitt-Hale et al., 2015). In contrast, CR, which is known to directly target and diminish IIS, has also been proven beneficial for both cognition and longevity in rodent models of Alzheimer's disease (Parrella et al., 2013). Figure 2 summarizes some of the NSC functions affected by the IIS pathway. As for mTOR, this highlights the need of a fine-tuned balance between IIS activation and inhibition throughout the lifespan. It is likely that several other factors such as oxidative state of the cell, biological age and brain region all play a role in this balance and can propel toward a positive or negative effect of the IIS pathway.

### Sirtuins

Sirtuins are a group of deacetylases initially shown to extend lifespan in yeast by regulating mitochondrial function and cellular redox state (Aguilaniu et al., 2003). Deacetylases are key regulatory proteins as they can control the expression of several genes. Furthermore, sirtuin activity is NAD-dependent, making them likely candidates for the molecular link between metabolism and aging owing to their ability to respond to the cell's energy status. Indeed, Sir2 activation in yeasts mimics CR-induced longevity, which in turn was shown to depend on the Sirtuin pathway (Lin et al., 2000). Increased lifespan as a result of sirtuin overexpression was also confirmed in other organisms such as *Drosophila* and *C. elegans* (Guarente, 2007). Similarly, the mammalian components of the sirtuin family, SIRT1 and SIRT4 have also been implicated in CR diet-regulated processes, showing that the link between metabolism and aging could be conserved across species (Guarente, 2007). Research into Sirtuins also highlighted the importance of NAMPT, the rate limiting enzyme in mammalian NAD+ synthesis; aging is accompanied by chronic DNA damage which leads to NAD+ depletion, Sirt1 inactivation and thus mitochondrial dysfunction (Guarente, 2014). Overexpression of NAMPT can rescue NAD+ levels and counteract these changes as shown by interesting studies investigating its effects on the accelerated aging disorder, Cockanye syndrome (van der Veer et al., 2007; Guarente, 2014; Scheibye-Knudsen et al., 2014). The importance of NAD+ and sirtuins in aging was also supported by recent studies by Song and colleagues showing that NAMPT inhibition is sufficient to induce senescence in human fibroblasts (Song et al., 2015).

The above studies however did not focus on the effects of NAMPT on NSCs. Interestingly, a recent study showed that NAMPT ablation recapitulated aspects of NSC aging such as decreased NSC proliferation in rodents (Stein and Imai, 2014), suggesting that NAMPT and the sirtuin pathway play key roles in NSC aging as well. Studies are now beginning to integrate the role of sirtuins, aging and diet or nutrition on the CNS specifically. For example, increased Sirt1 levels in murine brains due to CR were shown to increase anxiety and decrease exploratory drive (Libert et al., 2011). Whilst NSC function was not assessed in these studies, these conditions are known to involve deregulation of hippocampal neurogenesis (Libert et al., 2011; Hill et al., 2015) making it a plausible underlying mechanism. These studies also highlighted a possible disadvantage of increased Sirt1 activity and CR. In line with this, when the effect of SIRT1 deletion was investigated in prion disease, a condition in which neurogenic deregulation is also implicated (Gomez-Nicola et al., 2014), it was found to delay disease onset and to prolong the healthy portion of the affected animals. This was mimicked by CR (Chen et al., 2008). In contrast, others have shown that sirtuins can have neuroprotective functions in response to neuronal damage and neurodegenerative conditions; firstly, SIRT1 is upregulated in mouse models of Alzheimer's disease and amyotrophic lateral sclerosis and shown to enhance neuronal survival both *in vitro* and *in vivo* (Kim et al., 2007). Furthermore, SIRT1 was also found responsible for neuroprotective effects in murine models of axonal injury (Araki et al., 2004). SIRT1 overexpression was found to replicate the beneficial effects of CR in the context of several neurodegenerative conditions in various animal models (Gräff et al., 2013).

As many neurodegenerative conditions experience changes in neurogenesis, it is likely Sirt1 conveys some of its effects by influencing NSC function. SIRT1 activation in particular was linked to changes in neurogenesis in the perinatal SVZ; oxidizing conditions were found to activate SIRT1 and push the progenitor pool toward astrocytic differentiation whereas a reducing environment would promote neuronal differentiation (Prozorovski et al., 2008). Similarly, blocking Sirt1 activity disengaged redox changes from SVZ NSC fate (Prozorovski et al., 2008). Rafalski et al. in contrast, showed that Sirt1 inactivation pushed NSCs toward an oligodendrocyte lineage (Rafalski et al., 2013). Following Prozorovski's results, it is possible that the neuroprotective effect of SIRT1 activation, in part, is explained by improved CNS support from an increased number of astrocytes (See Table 1). An interesting avenue would be to investigate whether this finding is replicated in the DG and how this SIRT1-mediated modulation of the NSC pool changes with age.

Finally, these findings are now being investigated in human populations, Libert and colleagues for example showed that rare human SIRT1 variants are associated with anxiety and mood disorders (Libert et al., 2011). Interestingly, Sirt1 is also one of the two genes recently implicated in major depressive disorder by whole-genome sequencing findings (Cai et al., 2015). Though dietary interventions such as CR seem to be the most potent effectors of Sirtuin activation, some nutrients, like polyphenols have also been identified to directly activate components of this family (Howitz et al., 2003). Together the studies reported above, suggest the relationship between Sirt1, CR, and aging is regulated by intricate mechanisms, which become even more complex when acting upon different types of NSCs. The key NSC's functions acted upon by the sirtuin pathway are reported in Figure 2.

## Epigenetics

Epigenetics is at the forefront of aging research, a position supported by the recent establishment of an “epigenetic clock” by Steve Horvath, and has clear functions in adapting the organism's responses to its environment. These attributes make it a key mechanism mediating cellular and molecular responses to diet and aging processes (Rea et al., 2016). Epigenetic modulation comprises many mechanisms and is usually identified as the driving force behind changes in gene expression, which are not due to DNA sequence mutations. Such a process clearly has very important functions in stem cell regulation of different tissues as it can confer lineage-determining decisions as well as maintain quiescence. Epigenetics also plays an important role in aging; twin studies showed that genetic background only has a 25% influence on longevity (Herskind et al., 1996) suggesting that the remaining effects would be dictated by the environment, which usually ensues its effects through epigenetics. A role for epigenetics is also supported by notion that the effect of genetic variations on cognition and brain structure increases with age, recently reviewed by Papenberg et al. (2015). One of such environmental factors able to cause epigenetic changes is likely to be diet.

A line of thought believes that as epigenetic changes are less permanent, it would be more efficient to target and reverse them rather than targeting any genetic mutations which may arise as a result of aging (Rando and Chang, 2012; Beerman and Rossi, 2015). By restoring a “young” epigenetic environment one may be able to reverse age-related deficit; in a similar manner to the aforementioned heterochronic parabiosis studies (Villeda et al., 2014). Another factor supporting the notion that targeting epigenetics may be an efficient way to reverse aging comes from studies showing that only a small number of loci are altered consistently throughout aging, allowing for targeted interventions rather than global ones (Beerman and Rossi, 2015). Furthermore, epigenetic changes could be used as aging biomarkers, providing important information on the aging rate of an organism, with the potential to enable more precise preventive strategies. It is therefore important for us to understand how age and nutrition affect epigenetics and how this causes alterations in NSC regulation, disease progression and longevity. For the remainder of this section we will evaluate the evidence for such a relationship.

### Epigenetics and nutrition

There are several studies showing perinatal or *in utero* nutrition can have vast effect on health later in life, mainly related to cardiovascular disease, diabetes and obesity (Choi and Friso, 2010). Interestingly, these effects were also witnessed in the offspring of the affected animals suggesting that epigenetic changes could be passed through generations. The involvement of epigenetics was also confirmed via methylome analysis, though a causal link is yet to be shown (Radford et al., 2014; Fontana and Partridge, 2015). Such mechanisms are set in place to ensure adaptation for the fetus to its environment—CR and DR prepares a fetus to food scarcity whilst over nutrition primes for a nutrient abundant environment. Studies have suggested it may be the mismatching of predicted and actual nutrient availability to cause some of the detrimental health effects later in life (Perera and Herbstman, 2011).

While epigenetic changes occurring during the malleable stages development in response to nutrition have been extensively studied, those happening during adulthood are less known. The evidence from developmental studies reported above, however shows that mechanisms are set in place for epigenetics to respond to dietary and nutritional cues through the lifespan. Indeed, diet can alter epigenetics in several ways, these include the donation of methyl groups and the regulation of several enzymes (Mathers and Ford, 2009). As well as the alterations in response to CR and DR, other dietary alterations can have effects on epigenetics, such as the intake of specific nutrients. Studies on agouti mice showed a methyl-supplemented diet was able to cause DNA hypermethylation, making it likely for diet-acquired methyl donors like choline and methionine (an essential amino acid) to have similar effects (Waterland and Jirtle, 2003; Niculescu et al., 2006; Waterland et al., 2007). Furthermore, the trace mineral zinc interacts with histone deacetylase regulation (HDAC) and causes their inhibition (Myzak et al., 2006) whilst resveratrol, a dietary phenol, activates the HDAC SIRT1 (Rafalski and Brunet, 2011). Vitamin D also appears to form an important link between nutrition, aging and epigenetics as its varying concentrations can delay the aging phenotype in mice and its mechanism of action is known to involve histone acetylases (HATs) and HDACs (Tuohimaa, 2009).

### Epigenetics, aging, and neural stem cells

Several years ago it was found that cellular methyl content declines with increasing age in mammals (Wilson and Jones, 1983). This loss is likely to contribute to genomic instability, which is a hallmark of aging cells. Recently, Horvath and colleagues showed the existence of an epigenetic clock and developed a predictor able to estimate the methylation age of most tissues or cell types, suggesting that specific epigenetic changes occur as a result of age (Horvath, 2013).

Though there are a number of possible epigenetic marks, methylation is the mark considered to be the most stable one and thus the most likely candidate for encoding aging and nutritional changes. Methylation changes as a result of age can be divided into two main categories: those resulting from loss of fidelity when copying methylation marks and those arising from abnormal addition or removal of methylation marks. Further to this, a decrease in the activity and expression of the DNA methyltransferase DNMT1 was found with increasing age (Cooney, 1993). Interestingly, DNMT1 controls stem cell balance and lineage decisions in several tissues; its knock out in embryonic NSCs, for example, causes a preferential push toward astroglial differentiation (Fan et al., 2005). DNMT1 loss in general causes an aging phenotype highlighting it as a key molecule governing aging processes (Beerman and Rossi, 2015). In contrast, some housekeeping genes that are usually unmethylated seem to become methylated with age. This may be due to an increase in DNMT3 activity; DNMT3 is a methyltransferase responsible for de novo methylation, a process shown to be key in regulating stem cells as it halts self-renewal to allow differentiation. A DNMT3 knock out in mouse in fact showed impaired post-natal SVZ and SGL NSC differentiation (Wu et al., 2010) (See Table 1).

An important methylation mark, studied in the field of aging and NSCs, is the methylation of lysine 27 on histone 3 (H3K27). The differing methylation of this mark in fact can regulate adult NSC differentiation; when this mark has a single methylation, transcription is enabled, when the mark has 2 or 3 methyl groups gene transcription is repressed (Zhang et al., 2014). Another epigenetic mark often involved in aging is histone acetylation; several histone acetylases (HATs) are key in regulating the NSC pool by affecting both proliferation and differentiation of the neural progenitors. Sirtuins as detailed above are part of this family. There is also evidence of different epigenetic marks influencing one another—i.e., DNA methylation being restricted by acetylation marks (Beerman and Rossi, 2015). See Figure 2 for a representation of this interconnection. Mathers and Ford suggest that changes in methylation do not occur in every cell in a tissue. They explain how this leads to promoter methylation heterogeneity and thus to divergent gene expression and cellular response across different tissues with increasing age (Mathers and Ford, 2009). Together these studies show that epigenetic mechanisms are pivotal to a permissive or restricting environment for gene transcription in response to environmental cues and are therefore likely molecular effectors of nutrient intake and its ensuing effect on NSC regulation.

Finally, as well as sharing common downstream mechanisms, aging and nutrition can also affect one another; for example, nutrient intake may change as a result of age which could then cause the age-related epigenetic changes. A reduction of fruit and vegetable intake for instance, would reduce the intake of zinc and thus affect HDAC function (Mathers and Ford, 2009). Given the prominent role played by NSCs during aging, an exciting avenue would be to explore epigenetic changes in these cells in response to diet.

## Conclusion

In conclusion, though much progress has been made in establishing the role played by nutrition in longevity, and on stem cells more broadly, its role in NSCs regulation is still to be elucidated. In this review we have discussed how mTOR/IIS pathway inhibition and sirtuin activation may enhance longevity and CNS function, an effect achieved at least in part, through their impact on NSC function. Importantly, we have described how these responses can be shaped by diet and nutrition.

Whilst the positive impacts of CR and IF continue to be detailed in model systems, more targeted pharmacological approaches may be beneficial for use in frail and elderly populations. This highlights the need for a more thorough understanding of the molecular pathways involved in these dietary paradigms.

Further to this, much more research into the genetic and epigenetic influences of diet and nutrition is required, to refine populations that potentially stand to gain the most from such interventions. Despite these caveats, there is much excitement in the field as dietary paradigms such as IF are employed in human studies and the ensuing encouraging results with regards to their impact on cognitive performance (Brandhorst et al., 2015; Fontana and Partridge, 2015).

## Author contributions

The co-authors fulfill the criteria for authorship; they all contributed to the manuscript, approved it and agree to be accountable for its content. Cd, TM, and ST conceived the review, Cd carried out the literature review research, Cd and TM wrote the manuscript, ST revised the manuscript. The manuscript and parts of it, have not been, and will not be submitted elsewhere for publication.

## Funding

This review was written in relation to a grant awarded by the Medical Research Council UK (MR/N030087/1) (ST), Cd was supported by the Institute of Psychiatry, Psychology and Neuroscience Ph.D. Prize Studentship Award, TM was supported by the Medical Research Council UK (MR/K500811/1) and the Cohen Charitable Trust.

### Conflict of interest statement

The authors declare that the research was conducted in the absence of any commercial or financial relationships that could be construed as a potential conflict of interest.

## References

[B1] Åberg M. A. Åberg N. D. Hedbacker H. Oscarsson J. Eriksson P. S. (2000). Peripheral infusion of IGF-I selectively induces neurogenesis in the adult rat hippocampus. J. Neurosci. 20, 2896–2903. 10751442 10.1523/JNEUROSCI.20-08-02896.2000 PMC6772218

[B2] Åberg M. A. Åberg N. D. Palmer T. D. Alborn A.-M. Carlsson-Skwirut C. Bang P.. (2003). IGF-I has a direct proliferative effect in adult hippocampal progenitor cells. Mol. Cell. Neurosci. 24, 23–40. 10.1016/S1044-7431(03)00082-4 14550766

[B3] Aguilaniu H. Gustafsson L. Rigoulet M. Nyström T. (2003). Asymmetric inheritance of oxidatively damaged proteins during cytokinesis. Science 299, 1751–1753. 10.1126/science.1080418 12610228

[B4] Altman J. Das G. D. (1965). Autoradiographic and histological evidence of postnatal hippocampal neurogenesis in rats. J. Comp. Neurol. 124, 319–335. 10.1002/cne.901240303 5861717

[B5] Araki T. Sasaki Y. Milbrandt J. (2004). Increased nuclear NAD biosynthesis and SIRT1 activation prevent axonal degeneration. Science 305, 1010–1013. 10.1126/science.1098014 15310905

[B6] Arsenijevic Y. Weiss S. Schneider B. Aebischer P. (2001). Insulin-like growth factor-I is necessary for neural stem cell proliferation and demonstrates distinct actions of epidermal growth factor and fibroblast growth factor-2. J. Neurosci. 21, 7194–7202. 11549730 10.1523/JNEUROSCI.21-18-07194.2001 PMC6762999

[B7] Bartke A. Sun L. Y. Longo V. (2013). Somatotropic signaling: trade-offs between growth, reproductive development, and longevity. Physiol. Rev. 93, 571–598. 10.1152/physrev.00006.2012 23589828 PMC3768106

[B8] Beerman I. Rossi D. J. (2015). Epigenetic control of stem cell potential during homeostasis, aging, and disease. Cell Stem Cell 16, 613–625. 10.1016/j.stem.2015.05.009 26046761 PMC4469343

[B9] Behrens A. van Deursen J. M. Rudolph K. L. Schumacher B. (2014). Impact of genomic damage and ageing on stem cell function. Nat. Cell Biol. 16, 201–207. 10.1038/ncb2928 24576896 PMC4214082

[B10] Berg U. Bang P. (2004). Exercise and circulating insulin-like growth factor I. Horm. Res. 62(Suppl. 1), 50–58. 10.1159/000080759 15761233

[B11] Bergmann O. Spalding K. L. Frisén J. (2015). Adult neurogenesis in humans. Cold Spring Harb. Perspect. Biol. 7:a018994. 10.1101/cshperspect.a018994 26134318 PMC4484963

[B12] Blagosklonny M. V. (2008). Aging, stem cells, and mammalian target of rapamycin: a prospect of pharmacologic rejuvenation of aging stem cells. Rejuvenation Res. 11, 801–808. 10.1089/rej.2008.0722 18729812

[B13] Blagosklonny M. V. (2010). Calorie restriction: decelerating mTOR-driven aging from cells to organisms (including humans). Cell Cycle 9, 683–688. 10.4161/cc.9.4.10766 20139716

[B14] Blüher M. Kahn B. B. Kahn C. R. (2003). Extended longevity in mice lacking the insulin receptor in adipose tissue. Science 299, 572–574. 10.1126/science.1078223 12543978

[B15] Bonafè M. Barbieri M. Marchegiani F. Olivieri F. Ragno E. Giampieri C.. (2003). Polymorphic variants of insulin-like growth factor I (IGF-I) receptor and phosphoinositide 3-kinase genes affect IGF-I plasma levels and human longevity: cues for an evolutionarily conserved mechanism of life span control. J. Clin. Endocrinol. Metab. 88, 3299–3304. 10.1210/jc.2002-021810 12843179

[B16] Bondolfi L. Ermini F. Long J. M. Ingram D. K. Jucker M. (2004). Impact of age and caloric restriction on neurogenesis in the dentate gyrus of C57BL/6 mice. Neurobiol. Aging 25, 333–340. 10.1016/S0197-4580(03)00083-6 15123339

[B17] Bracko O. Singer T. Aigner S. Knobloch M. Winner B. Ray J.. (2012). Gene expression profiling of neural stem cells and their neuronal progeny reveals IGF2 as a regulator of adult hippocampal neurogenesis. J. Neurosci. 32, 3376–3387. 10.1523/JNEUROSCI.4248-11.2012 22399759 PMC3338187

[B18] Brandhorst S. Choi I. Y. Wei M. Cheng C. W. Sedrakyan S. Navarrete G.. (2015). A periodic diet that mimics fasting promotes multi-system regeneration, enhanced cognitive performance, and healthspan. Cell Metab. 22, 86–99. 10.1016/j.cmet.2015.05.012 26094889 PMC4509734

[B19] Cai N. Bigdeli T. B. Kretzschmar W. Li Y. Liang J. Song L.. (2015). Sparse whole-genome sequencing identifies two loci for major depressive disorder. Nature 523, 588–591. 10.1038/nature14659 26176920 PMC4522619

[B20] Carson M. J. Behringer R. R. Brinster R. L. McMorris F. A. (1993). Insulin-like growth factor I increases brain growth and central nervous system myelination in tTransgenic mice. Neuron 10, 729–740. 10.1016/0896-6273(93)90173-O 8386530

[B21] Castilho R. M. Squarize C. H. Chodosh L. A. Williams B. O. Gutkind J. S. (2009). mTOR mediates Wnt-induced epidermal stem cell exhaustion and aging. Cell Stem Cell 5, 279–289. 10.1016/j.stem.2009.06.017 19733540 PMC2939833

[B22] Chaker Z. Aïd S. Berry H. Holzenberger M. (2015). Suppression of IGF-I signals in neural stem cells enhances neurogenesis and olfactory function during aging. Aging Cell 14, 847–856. 10.1111/acel.12365 26219530 PMC4568972

[B23] Check Hayden E. (2014). Pet dogs set to test anti-ageing drug. Nature 514, 546. 10.1038/514546a 25355339

[B24] Chen D. Steele A. D. Hutter G. Bruno J. Govindarajan A. Easlon E.. (2008). The role of calorie restriction and SIRT1 in prion-mediated neurodegeneration. Exp. Gerontol. 43, 1086–1093. 10.1016/j.exger.2008.08.050 18799131 PMC2735260

[B25] Chen D. Y. Stern S. A. Garcia-Osta A. Saunier-Rebori B. Pollonini G. Bambah-Mukku D.. (2011). A critical role for IGF-II in memory consolidation and enhancement. Nature 469, 491–497. 10.1038/nature09667 21270887 PMC3908455

[B26] Chigogora S. Zaninotto P. Kivimaki M. Steptoe A. Batty G. D. (2016). Insulin-like growth factor 1 and risk of depression in older people: the English Longitudinal Study of Ageing. Transl. Psychiatry 6:e898. 10.1038/tp.2016.167 27648920 PMC5048205

[B27] Choi S.-W. Friso S. (2010). Epigenetics: a new bridge between nutrition and health. Adv. Nutr. 1, 8–16. 10.3945/an.110.1004 22043447 PMC3042783

[B28] Clayton R. N. (2003). Cardiovascular function in acromegaly. Endocr. Rev. 24, 272–277. 10.1210/er.2003-0009 12788799

[B29] Conboy I. M. Rando T. A. (2012). Heterochronic parabiosis for the study of the effects of aging on stem cells and their niches. Cell Cycle 11, 2260–2267. 10.4161/cc.20437 22617385 PMC3383588

[B30] Cooney C. A. (1993). Are somatic cells inherently deficient in methylation metabolism? A proposed mechanism for DNA methylation loss, senescence and aging. Growth. Dev. Aging 57, 261–273. 8300279

[B31] Cuervo A. M. (2008). Autophagy and aging: keeping that old broom working. Trends Genet. 24, 604–612. 10.1016/j.tig.2008.10.002 18992957 PMC2745226

[B32] Day K. Shefer G. Shearer A. Yablonka-Reuveni Z. (2010). The depletion of skeletal muscle satellite cells with age is concomitant with reduced capacity of single progenitors to produce reserve progeny. Dev. Biol. 340, 330–343. 10.1016/j.ydbio.2010.01.006 20079729 PMC2854302

[B33] Enwere E. Shingo T. Gregg C. Fujikawa H. Ohta S. Weiss S. (2004). Aging results in reduced epidermal growth factor receptor signaling, diminished olfactory neurogenesis, and deficits in fine olfactory discrimination. J. Neurosci. 24, 8354–8365. 10.1523/JNEUROSCI.2751-04.2004 15385618 PMC6729689

[B34] Ernst A. Alkass K. Bernard S. Salehpour M. Perl S. Tisdale J.. (2014). Neurogenesis in the striatum of the adult human brain. Cell 156, 1072–1083. 10.1016/j.cell.2014.01.044 24561062

[B35] Fan G. Martinowich K. Chin M. H. He F. Fouse S. D. Hutnick L.. (2005). DNA methylation controls the timing of astrogliogenesis through regulation of JAK-STAT signaling. Development 132, 3345–3356. 10.1242/dev.01912 16014513

[B36] Fok W. C. Chen Y. Bokov A. Zhang Y. Salmon A. B. Diaz V.. (2014). Mice fed rapamycin have an increase in lifespan associated with major changes in the liver transcriptome. PLoS ONE 9:e83988. 10.1371/journal.pone.0083988 24409289 PMC3883653

[B37] Fontana L. Partridge L. (2015). Promoting health and longevity through diet: from model organisms to humans. Cell 161, 106–118. 10.1016/j.cell.2015.02.020 25815989 PMC4547605

[B38] Fontana L. Partridge L. Longo V. D. (2010). Extending healthy life span–from yeast to humans. Science 328, 321–326. 10.1126/science.1172539 20395504 PMC3607354

[B39] Franklin N. C. Tate C. A. (2008). Lifestyle and successful aging: an overview. Am. J. Lifestyle Med. 3, 6–11. 10.1177/1559827608326125

[B40] Gems D. de la Guardia Y. (2013). Alternative perspectives on aging in caenorhabditis elegans: reactive oxygen species or hyperfunction? Antioxid. Redox Signal. 19, 321–329. 10.1089/ars.2012.4840 22870907 PMC5395017

[B41] Gomez-Nicola D. Suzzi S. Vargas-Caballero M. Fransen N. L. Al-Malki H. Cebrian-Silla A.. (2014). Temporal dynamics of hippocampal neurogenesis in chronic neurodegeneration. Brain 137, 2312–2328. 10.1093/brain/awu155 24941947 PMC4107745

[B42] Gräff J. Kahn M. Samiei A. Gao J. Ota K. T. Rei D.. (2013). A dietary regimen of caloric restriction or pharmacological activation of SIRT1 to delay the onset of neurodegeneration. J. Neurosci. 33, 8951–8960. 10.1523/JNEUROSCI.5657-12.2013 23699506 PMC3775567

[B43] Guarente L. (2007). Sirtuins in aging and disease. Cold Spring Harb. Symp. Quant. Biol. 72, 483–488. 10.1101/sqb.2007.72.024 18419308

[B44] Guarente L. (2014). Linking DNA damage, NAD(+)/SIRT1, and aging. Cell Metab. 20, 706–707. 10.1016/j.cmet.2014.10.015 25440052

[B45] Han J. Wang B. Xiao Z. Gao Y. Zhao Y. Zhang J.. (2008). Mammalian target of rapamycin (mTOR) is involved in the neuronal differentiation of neural progenitors induced by insulin. Mol. Cell. Neurosci. 39, 118–124. 10.1016/j.mcn.2008.06.003 18620060

[B46] Harrison D. E. Strong R. Sharp Z. D. Nelson J. F. Astle C. M. Flurkey K.. (2009). Rapamycin fed late in life extends lifespan in genetically heterogeneous mice. Nature 460, 392–395. 10.1038/nature08221 19587680 PMC2786175

[B47] Herskind A. M. McGue M. Holm N. V. Sørensen T. I. Harvald B. Vaupel J. W. (1996). The heritability of human longevity: a population-based study of 2872 Danish twin pairs born 1870-1900. Hum. Genet. 97, 319–323. 10.1007/BF02185763 8786073

[B48] Hill A. S. Sahay A. Hen R. (2015). Increasing adult hippocampal neurogenesis is sufficient to reduce anxiety and depression-like behaviors. Neuropsychopharmacology 40, 2368–2378. 10.1038/npp.2015.85 25833129 PMC4538351

[B49] Horvath S. (2013). DNA methylation age of human tissues and cell types. Genome Biol. 14:R115. 10.1186/gb-2013-14-10-r115 24138928 PMC4015143

[B50] Howitz K. T. Bitterman K. J. Cohen H. Y. Lamming D. W. Lavu S. Wood J. G.. (2003). Small molecule activators of sirtuins extend *Saccharomyces cerevisiae* lifespan. Nature 425, 191–196. 10.1038/nature01960 12939617

[B51] Ingram D. K. Weindruch R. Spangler E. L. Freeman J. R. Walford R. L. (1987). Dietary restriction benefits learning and motor performance of aged mice. J. Gerontol. 42, 78–81. 10.1093/geronj/42.1.78 3794202

[B52] Itoh T. Imano M. Nishida S. Tsubaki M. Mizuguchi N. Hashimoto S.. (2012). (−)-Epigallocatechin-3-gallate increases the number of neural stem cells around the damaged area after rat traumatic brain injury. J. Neural Transm. 119, 877–890. 10.1007/s00702-011-0764-9 22212485

[B53] Kalichman L. Rodrigues B. Gurvich D. Israelov Z. Spivak E. (2007). Impact of patient's weight on stroke rehabilitation results. Am. J. Phys. Med. Rehabil. 86, 650–655. 10.1097/PHM.0b013e318115f41b 17667195

[B54] Katsimpardi L. Litterman N. K. Schein P. A. Miller C. M. Loffredo F. S. Wojtkiewicz G. R.. (2014). Vascular and neurogenic rejuvenation of the aging mouse brain by young systemic factors. Science 344, 630–634. 10.1126/science.1251141 24797482 PMC4123747

[B55] Keenan K. P. Smith P. F. Hertzog P. Soper K. Ballam G. C. Clark R. L. (1994). The effects of overfeeding and dietary restriction on Sprague-Dawley rat survival and early pathology biomarkers of aging. Toxicol. Pathol. 22, 300–315. 10.1177/019262339402200308 7817120

[B56] Kim D. Nguyen M. D. Dobbin M. M. Fischer A. Sananbenesi F. Rodgers J. T.. (2007). SIRT1 deacetylase protects against neurodegeneration in models for Alzheimer's disease and amyotrophic lateral sclerosis. EMBO J. 26, 3169–3179. 10.1038/sj.emboj.7601758 17581637 PMC1914106

[B57] Kimura K. D. Tissenbaum H. A. Liu Y. Ruvkun G. (1997). daf-2, an insulin receptor-like gene that regulates longevity and diapause in *Caenorhabditis elegans*. Science 277, 942–946. 10.1126/science.277.5328.942 9252323

[B58] Kojima T. Kamei H. Aizu T. Arai Y. Takayama M. Nakazawa S.. (2004). Association analysis between longevity in the Japanese population and polymorphic variants of genes involved in insulin and insulin-like growth factor 1 signaling pathways. Exp. Gerontol. 39, 1595–1598. 10.1016/j.exger.2004.05.007 15582274

[B59] Kokoeva M. V. Yin H. Flier J. S. (2005). Neurogenesis in the hypothalamus of adult mice: potential role in energy balance. Science 310, 679–683. 10.1126/science.1115360 16254185

[B60] Kumar S. Parkash J. Kataria H. Kaur G. (2009). Interactive effect of excitotoxic injury and dietary restriction on neurogenesis and neurotrophic factors in adult male rat brain. Neurosci. Res. 65, 367–374. 10.1016/j.neures.2009.08.015 19732799

[B61] Lee J. Duan W. Long J. M. Ingram D. K. Mattson M. P. (2000). Dietary restriction increases the number of newly generated neural cells, and induces BDNF expression, in the dentate gyrus of rats. J. Mol. Neurosci. 15, 99–108. 10.1385/JMN:15:2:99 11220789

[B62] Lee J. Seroogy K. B. Mattson M. P. (2002). Dietary restriction enhances neurotrophin expression and neurogenesis in the hippocampus of adult mice. J. Neurochem. 80, 539–547. 10.1046/j.0022-3042.2001.00747.x 11905999

[B63] Lehtinen M. K. Zappaterra M. W. Chen X. Yang Y. J. Hill A. D. Lun M.. (2011). The cerebrospinal fluid provides a proliferative niche for neural progenitor cells. Neuron 69, 893–905. 10.1016/j.neuron.2011.01.023 21382550 PMC3085909

[B64] Libert S. Pointer K. Bell E. L. Das A. Cohen D. E. Asara J. M.. (2011). SIRT1 activates MAO-A in the brain to mediate anxiety and exploratory drive. Cell 147, 1459–1472. 10.1016/j.cell.2011.10.054 22169038 PMC3443638

[B65] Lin S. J. Defossez P. A. Guarente L. (2000). Requirement of NAD and SIR2 for life-span extension by calorie restriction in *Saccharomyces cerevisiae*. Science 289, 2126–2128. 10.1126/science.289.5487.2126 11000115

[B66] Loffredo F. S. Steinhauser M. L. Jay S. M. Gannon J. Pancoast J. R. Yalamanchi P.. (2013). Growth differentiation factor 11 is a circulating factor that reverses age-related cardiac hypertrophy. Cell 153, 828–839. 10.1016/j.cell.2013.04.015 23663781 PMC3677132

[B67] López-Otín C. Blasco M. A. Partridge L. Serrano M. Kroemer G. (2013). The hallmarks of aging. Cell 153, 1194–1217. 10.1016/j.cell.2013.05.039 23746838 PMC3836174

[B68] López-Toledano M. A. Shelanski M. L. (2004). Neurogenic effect of beta-amyloid peptide in the development of neural stem cells. J. Neurosci. 24, 5439–5444. 10.1523/JNEUROSCI.0974-04.2004 15190117 PMC6729298

[B69] López-Toledano M. A. Shelanski M. L. (2007). Increased neurogenesis in young transgenic mice overexpressing human APP(Sw, Ind). J. Alzheimers. Dis. 12, 229–240. 18057556 10.3233/jad-2007-12304

[B70] Magri L. Galli R. (2013). mTOR signaling in neural stem cells: from basic biology to disease. Cell. Mol. Life Sci. 70, 2887–2898. 10.1007/s00018-012-1196-x 23124271 PMC11113161

[B71] Martínez-Cisuelo V. Gómez J. García-Junceda I. Naudí A. Cabré R. Mota-Martorell N.. (2016). Rapamycin reverses age-related increases in mitochondrial ROS production at complex I, oxidative stress, accumulation of mtDNA fragments inside nuclear DNA, and lipofuscin level, and increases autophagy, in the liver of middle-aged mice. Exp. Gerontol. 83, 130–138. 10.1016/j.exger.2016.08.002 27498120

[B72] Maruszak A. Pilarski A. Murphy T. Branch N. Thuret S. (2014). Hippocampal neurogenesis in Alzheimer's disease: is there a role for dietary modulation? J. Alzheimers. Dis. 38, 11–38. 10.3233/JAD-131004 23948932

[B73] Mathers J. C. Ford D. (2009). Nutrition, epigenetics, and ageing, in Nutrients and Epigenetics, eds Choi S.-W. Frisio S. (Boca Raton, FL: CRC Press; Taylor & Francis Group), 175–196. 10.1201/9781420063561.ch8

[B74] Mattson M. P. (2012). Energy intake and exercise as determinants of brain health and vulnerability to injury and disease. Cell Metab. 16, 706–722. 10.1016/j.cmet.2012.08.012 23168220 PMC3518570

[B75] Mihaylova M. M. Sabatini D. M. Yilmaz Ö. H. (2014). Dietary and metabolic control of stem cell function in physiology and cancer. Cell Stem Cell 14, 292–305. 10.1016/j.stem.2014.02.008 24607404 PMC3992244

[B76] Murphy T. Thuret S. (2015). The systemic milieu as a mediator of dietary influence on stem cell function during ageing. Ageing Res. Rev. 19, 53–64. 10.1016/j.arr.2014.11.004 25481406

[B77] Myzak M. C. Ho E. Dashwood R. H. (2006). Dietary agents as histone deacetylase inhibitors. Mol. Carcinog. 45, 443–446. 10.1002/mc.20224 16652377 PMC2267873

[B78] Nagai M. Kuriyama S. Kakizaki M. Ohmori-Matsuda K. Sone T. Hozawa A.. (2012). Impact of obesity, overweight and underweight on life expectancy and lifetime medical expenditures: the Ohsaki Cohort Study. BMJ Open 2:e000940. 10.1136/bmjopen-2012-000940 22581797 PMC3353127

[B79] Niccoli T. Partridge L. (2012). Ageing as a risk factor for disease. Curr. Biol. 22, R741–R752. 10.1016/j.cub.2012.07.024 22975005

[B80] Niculescu M. D. Craciunescu C. N. Zeisel S. H. (2006). Dietary choline deficiency alters global and gene-specific DNA methylation in the developing hippocampus of mouse fetal brains. FASEB J. 20, 43–49. 10.1096/fj.05-4707com 16394266 PMC1635129

[B81] O' Neill C. (2013). PI3-kinase/Akt/mTOR signaling: impaired on/off switches in aging, cognitive decline and Alzheimer's disease. Exp. Gerontol. 48, 647–653. 10.1016/j.exger.2013.02.025 23470275

[B82] Ochocki J. D. Simon M. C. (2013). Nutrient-sensing pathways and metabolic regulation in stem cells. J. Cell Biol. 203, 23–33. 10.1083/jcb.201303110 24127214 PMC3798256

[B83] Paik J. Ding Z. Narurkar R. Ramkissoon S. Muller F. Kamoun W. S.. (2009). FoxOs cooperatively regulate diverse pathways governing neural stem cell homeostasis. Cell Stem Cell 5, 540–553. 10.1016/j.stem.2009.09.013 19896444 PMC3285492

[B84] Paliouras G. Hamilton L. Aumont A. Joppé S. Barnabé-Heider F. Fernandes K. (2012). Mammalian target of rapamycin signaling is a key regulator of the transit-amplifying progenitor pool in the adult and aging forebrain. J. Neurosci. 32, 15012–15026. 10.1523/JNEUROSCI.2248-12.2012 23100423 PMC6704835

[B85] Papenberg G. Lindenberger U. Bäckman L. (2015). Aging-related magnification of genetic effects on cognitive and brain integrity. Trends Cogn. Sci. 19, 506–514. 10.1016/j.tics.2015.06.008 26187033

[B86] Park D. Yang Y.-H. Bae D. K. Lee S. H. Yang G. Kyung J.. (2013). Improvement of cognitive function and physical activity of aging mice by human neural stem cells over-expressing choline acetyltransferase. Neurobiol. Aging 34, 2639–2646. 10.1016/j.neurobiolaging.2013.04.026 23731954

[B87] Parrella E. Maxim T. Maialetti F. Zhang L. Wan J. Wei M.. (2013). Protein restriction cycles reduce IGF-1 and phosphorylated Tau, and improve behavioral performance in an Alzheimer's disease mouse model. Aging Cell 12, 257–268. 10.1111/acel.12049 23362919 PMC3982836

[B88] Pencea V. Bingaman K. D. Freedman L. J. Luskin M. B. (2001). Neurogenesis in the subventricular zone and rostral migratory stream of the neonatal and adult primate forebrain. Exp. Neurol. 172, 1–16. 10.1006/exnr.2001.7768 11681836

[B89] Perera F. Herbstman J. (2011). Prenatal environmental exposures, epigenetics, and disease. Reprod. Toxicol. 31, 363–373. 10.1016/j.reprotox.2010.12.055 21256208 PMC3171169

[B90] Prozorovski T. Schulze-Topphoff U. Glumm R. Baumgart J. Schröter F. Ninnemann O.. (2008). Sirt1 contributes critically to the redox-dependent fate of neural progenitors. Nat. Cell Biol. 10, 385–394. 10.1038/ncb1700 18344989

[B91] Radford E. J. Ito M. Shi H. Corish J. A. Yamazawa K. Isganaitis E.. (2014). *In utero* undernourishment perturbs the adult sperm methylome and intergenerational metabolism. Science 345:1255903. 10.1126/science.1255903 25011554 PMC4404520

[B92] Rafalski V. A. Brunet A. (2011). Energy metabolism in adult neural stem cell fate. Prog. Neurobiol. 93, 182–203. 10.1016/j.pneurobio.2010.10.007 21056618

[B93] Rafalski V. A. Ho P. P. Brett J. O. Ucar D. Dugas J. C. Pollina E. A.. (2013). Expansion of oligodendrocyte progenitor cells following SIRT1 inactivation in the adult brain. Nat. Cell Biol. 15, 614–624. 10.1038/ncb2735 23644469 PMC4026158

[B94] Rafalski V. A. Mancini E. Brunet A. (2012). Energy metabolism and energy-sensing pathways in mammalian embryonic and adult stem cell fate. J. Cell Sci. 125, 5597–5608. 10.1242/jcs.114827 23420198 PMC3575699

[B95] Rando T. A. Chang H. Y. (2012). Aging, rejuvenation, and epigenetic reprogramming: resetting the aging clock. Cell 148, 46–57. 10.1016/j.cell.2012.01.003 22265401 PMC3336960

[B96] Rea I. M. Dellet M. Mills K. I. The ACUME2 Project. (2016). Living long and ageing well: is epigenomics the missing link between nature and nurture? Biogerontology 17, 33–54. 10.1007/s10522-015-9589-5 26133292

[B97] Reed M. J. Penn P. E. Li Y. Birnbaum R. Vernon R. B. Johnson T. S.. (1996). Enhanced cell proliferation and biosynthesis mediate improved wound repair in refed, caloric-restricted mice. Mech. Ageing Dev. 89, 21–43. 10.1016/0047-6374(96)01737-X 8819104

[B98] Renault V. M. Rafalski V. A. Morgan A. A. Salih D. A. M. Brett J. O. Webb A. E.. (2009). FoxO3 regulates neural stem cell homeostasis. Cell Stem Cell 5, 527–539. 10.1016/j.stem.2009.09.014 19896443 PMC2775802

[B99] Romine J. Gao X. Xu X.-M. So K. F. Chen J. (2015). The proliferation of amplifying neural progenitor cells is impaired in the aging brain and restored by the mTOR pathway activation. Neurobiol. Aging 36, 1716–1726. 10.1016/j.neurobiolaging.2015.01.003 25655438

[B100] Sato A. Sunayama J. Matsuda K. Tachibana K. Sakurada K. Tomiyama A.. (2010). Regulation of neural stem/progenitor cell maintenance by PI3K and mTOR. Neurosci. Lett. 470, 115–120. 10.1016/j.neulet.2009.12.067 20045038

[B101] Scheibye-Knudsen M. Mitchell S. J. Fang E. F. Iyama T. Ward T. Wang J.. (2014). A high-fat diet and NAD(+) activate Sirt1 to rescue premature aging in cockayne syndrome. Cell Metab. 20, 840–855. 10.1016/j.cmet.2014.10.005 25440059 PMC4261735

[B102] Shukitt-Hale B. Bielinski D. F. Lau F. C. Willis L. M. Carey A. N. Joseph J. A.. (2015). The beneficial effects of berries on cognition, motor behaviour and neuronal function in ageing. Br. J. Nutr. 114, 1542–1549. 10.1017/S0007114515003451 26392037

[B103] Signer R. A. J. Morrison S. J. (2013). Mechanisms that regulate stem cell aging and life span. Cell Stem Cell 12, 152–165. 10.1016/j.stem.2013.01.001 23395443 PMC3641677

[B104] Solon-Biet S. M. McMahon A. C. Ballard J. W. O. Ruohonen K. Wu L. E. Cogger V. C. (2014). The ratio of macronutrients, not caloric intake, dictates cardiometabolic health, aging, and longevity in *ad libitum*-fed mice. Cell Metab. 19, 418–430. 10.1016/j.cmet.2014.02.009 24606899 PMC5087279

[B105] Solon-Biet S. M. Mitchell S. J. Coogan S. C. P. Cogger V. C. Gokarn R. McMahon A. C.. (2015). Dietary protein to carbohydrate ratio and caloric restriction: comparing metabolic outcomes in mice. Cell Rep. 11, 1529–1534. 10.1016/j.celrep.2015.05.007 26027933 PMC4472496

[B106] Song T.-Y. Yeh S.-L. Hu M.-L. Chen M.-Y. Yang N.-C. (2015). A Nampt inhibitor FK866 mimics vitamin B3 deficiency by causing senescence of human fibroblastic Hs68 cells via attenuation of NAD(+)-SIRT1 signaling. Biogerontology 16, 789–800. 10.1007/s10522-015-9605-9 26330291

[B107] Spalding K. L. Bergmann O. Alkass K. Bernard S. Salehpour M. Huttner H. B.. (2013). Dynamics of hippocampal neurogenesis in adult humans. Cell 153, 1219–1227. 10.1016/j.cell.2013.05.002 23746839 PMC4394608

[B108] Stangl D. Thuret S. (2009). Impact of diet on adult hippocampal neurogenesis. Genes Nutr. 4, 271–282. 10.1007/s12263-009-0134-5 19685256 PMC2775886

[B109] Stein L. R. Imai S. (2014). Specific ablation of Nampt in adult neural stem cells recapitulates their functional defects during aging. EMBO J. 33, 1321–1340. 10.1002/embj.201386917 24811750 PMC4194122

[B110] Steinmetz A. B. Johnson S. A. Iannitelli D. E. Pollonini G. Alberini C. M. (2016). Insulin-like growth factor 2 rescues aging-related memory loss in rats. Neurobiol. Aging 44, 9–21. 10.1016/j.neurobiolaging.2016.04.006 27318130 PMC4913033

[B111] Suh Y. Atzmon G. Cho M.-O. Hwang D. Liu B. Leahy D. J.. (2008). Functionally significant insulin-like growth factor I receptor mutations in centenarians. Proc. Natl. Acad. Sci. U.S.A. 105, 3438–3442. 10.1073/pnas.0705467105 18316725 PMC2265137

[B112] Taguchi A. Wartschow L. M. White M. F. (2007). Brain IRS2 signaling coordinates life span and nutrient homeostasis. Science 317, 369–372. 10.1126/science.1142179 17641201

[B113] Tan P. Wang Y.-J. Li S. Wang Y. He J.-Y. Chen Y.-Y.. (2016). The PI3K/Akt/mTOR pathway regulates the replicative senescence of human VSMCs. Mol. Cell. Biochem. 422, 1–10. 10.1007/s11010-016-2796-9 27619662

[B114] Teumer A. Qi Q. Nethander M. Aschard H. Bandinelli S. Beekman M.. (2016). Genomewide meta-analysis identifies loci associated with IGF-I and IGFBP-3 levels with impact on age-related traits. Aging Cell 15, 811–824. 10.1111/acel.12490 27329260 PMC5013013

[B115] Tuohimaa P. (2009). Vitamin D and aging. J. Steroid Biochem. Mol. Biol. 114, 78–84. 10.1016/j.jsbmb.2008.12.020 19444937

[B116] van der Spoel E. Jansen S. W. Akintola A. A. Ballieux B. E. Cobbaert C. M. Slagboom P. E. (2016). Growth hormone secretion is diminished and tightly controlled in humans enriched for familial longevity. Aging Cell 15, 1126–1131. 10.1111/acel.12519 PMC6398524 27605408

[B117] van der Veer E. Ho C. O'Neil C. Barbosa N. Scott R. Cregan S. P.. (2007). Extension of human cell lifespan by nicotinamide phosphoribosyltransferase. J. Biol. Chem. 282, 10841–10845. 10.1074/jbc.C700018200 17307730

[B118] van Heemst D. (2010). Insulin, IGF-1 and longevity. Aging Dis. 1, 147–157. 22396862 PMC3295030

[B119] Verburgh K. (2015). Nutrigerontology: why we need a new scientific discipline to develop diets and guidelines to reduce the risk of aging-related diseases. Aging Cell 14, 17–24. 10.1111/acel.12284 25470422 PMC4326913

[B120] Villeda S. A. Luo J. Mosher K. I. Zou B. Britschgi M. Bieri G.. (2011). The ageing systemic milieu negatively regulates neurogenesis and cognitive function. Nature 477, 90–94. 10.1038/nature10357 21886162 PMC3170097

[B121] Villeda S. A. Plambeck K. E. Middeldorp J. Castellano J. M. Mosher K. I. Luo J.. (2014). Young blood reverses age-related impairments in cognitive function and synaptic plasticity in mice. Nat. Med. 20, 659–663. 10.1038/nm.3569 24793238 PMC4224436

[B122] Wanke V. Cameroni E. Uotila A. Piccolis M. Urban J. Loewith R.. (2008). Caffeine extends yeast lifespan by targeting TORC1. Mol. Microbiol. 69, 277–285. 10.1111/j.1365-2958.2008.06292.x 18513215

[B123] Warr M. R. Binnewies M. Flach J. Reynaud D. Garg T. Malhotra R.. (2013). FOXO3A directs a protective autophagy program in haematopoietic stem cells. Nature 494, 323–327. 10.1038/nature11895 23389440 PMC3579002

[B124] Waterland R. A. Jirtle R. L. (2003). Transposable elements: targets for early nutritional effects on epigenetic gene regulation. Mol. Cell. Biol. 23, 5293–5300. 10.1128/MCB.23.15.5293-5300.2003 12861015 PMC165709

[B125] Waterland R. A. Travisano M. Tahiliani K. G. (2007). Diet-induced hypermethylation at agouti viable yellow is not inherited transgenerationally through the female. FASEB J. 21, 3380–3385. 10.1096/fj.07-8229com 17551099

[B126] Wilson V. Jones P. (1983). DNA methylation decreases in aging but not in immortal cells. Science 220, 1055–1057. 10.1126/science.6844925 6844925

[B127] Winner B. Kohl Z. Gage F. H. (2011). Neurodegenerative disease and adult neurogenesis. Eur. J. Neurosci. 33, 1139–1151. 10.1111/j.1460-9568.2011.07613.x 21395858

[B128] Witte A. V. Fobker M. Gellner R. Knecht S. Flöel A. (2009). Caloric restriction improves memory in elderly humans. Proc. Natl. Acad. Sci. U.S.A. 106, 1255–1260. 10.1073/pnas.0808587106 19171901 PMC2633586

[B129] Wu H. Coskun V. Tao J. Xie W. Ge W. Yoshikawa K.. (2010). Dnmt3a-dependent nonpromoter DNA methylation facilitates transcription of neurogenic genes. Science 329, 444–448. 10.1126/science.1190485 20651149 PMC3539760

[B130] Yang T.-T. Lo C.-P. Tsai P.-S. Wu S.-Y. Wang T.-F. Chen Y.-W.. (2015). Aging and exercise affect hippocampal neurogenesis via different mechanisms. PLoS ONE 10:e0132152. 10.1371/journal.pone.0132152 26147302 PMC4493040

[B131] Ye P. Li L. Richards R. G. DiAugustine R. P. D'Ercole A. J. (2002). Myelination is altered in insulin-like growth factor-I null mutant mice. J. Neurosci. 22, 6041–6051. 12122065 10.1523/JNEUROSCI.22-14-06041.2002 PMC6757955

[B132] Yu S.-W. Baek S.-H. Brennan R. T. Bradley C. J. Park S. K. Lee Y. S.. (2008). Autophagic death of adult hippocampal neural stem cells following insulin withdrawal. Stem Cells 26, 2602–2610. 10.1634/stemcells.2008-0153 18653772

[B133] Yuan R. Tsaih S.-W. Petkova S. B. Marin de Evsikova C. Xing S. Marion M. A.. (2009). Aging in inbred strains of mice: study design and interim report on median lifespans and circulating IGF1 levels. Aging Cell 8, 277–287. 10.1111/j.1474-9726.2009.00478.x 19627267 PMC2768517

[B134] Zhang J. Ji F. Liu Y. Lei X. Li H. Ji G.. (2014). Ezh2 regulates adult hippocampal neurogenesis and memory. J. Neurosci. 34, 5184–5199. 10.1523/JNEUROSCI.4129-13.2014 24719098 PMC6609005

[B135] Ziegler A. N. Levison S. W. Wood T. L. (2015). Insulin and IGF receptor signalling in neural-stem-cell homeostasis. Nat. Rev. Endocrinol. 11, 161–170. 10.1038/nrendo.2014.208 25445849 PMC5513669

[B136] Ziegler A. N. Schneider J. S. Qin M. Tyler W. A. Pintar J. E. Fraidenraich D.. (2012). IGF-II promotes stemness of neural restricted precursors. Stem Cells 30, 1265–1276. 10.1002/stem.1095 22593020 PMC5581406

